# COVID-19 Vaccines and Myocarditis: An Overview of Current Evidence

**DOI:** 10.3390/biomedicines11051469

**Published:** 2023-05-17

**Authors:** Altijana Hromić-Jahjefendić, Abas Sezer, Alaa A. A. Aljabali, Ángel Serrano-Aroca, Murtaza M. Tambuwala, Vladimir N. Uversky, Elrashdy M. Redwan, Debmalya Barh, Kenneth Lundstrom

**Affiliations:** 1 Department of Genetics and Bioengineering, Faculty of Engineering and Natural Sciences, International University of Sarajevo, Hrasnicka cesta 15, 71000 Sarajevo, Bosnia and Herzegovina; asezer@ius.edu.ba; 2 Department of Pharmaceutics and Pharmaceutical Technology, Faculty of Pharmacy, Yarmouk University, P.O. Box 566, Irbid 21163, Jordan; alaaj@yu.edu.jo; 3 Biomaterials and Bioengineering Laboratory, Centro de Investigación Traslacional San Alberto Magno, Universidad Católica de Valencia San Vicente Mártir, c/Guillem de Castro 94, 46001 Valencia, Spain; angel.serrano@ucv.es; 4 Lincoln Medical School, Brayford Pool Campus, University of Lincoln, Lincoln LN6 7TS, UK; mtambuwala@lincoln.ac.uk; 5 Department of Molecular Medicine and USF Health Byrd Alzheimer’s Institute, Morsani College of Medicine, University of South Florida, Tampa, FL 33612, USA; vuversky@usf.edu; 6 Department of Biological Science, Faculty of Science, King Abdulaziz University, Jeddah 21589, Saudi Arabia; lradwan@kau.edu.sa; 7 Therapeutic and Protective Proteins Laboratory, Protein Research Department, Genetic Engineering and Biotechnology Research Institute, City for Scientific Research and Technology Applications, New Borg EL-Arab 21934, Egypt; 8 Institute of Integrative Omics and Applied Biotechnology (IIOAB), Nonakuri, Purba Medinipur 721172, India; dr.barh@gmail.com; 9 Department of Genetics, Ecology and Evolution, Institute of Biological Sciences, Federal University of Minas Gerais, Belo Horizonte 31270-901, Brazil; 10 Pan Therapeutics, Route de Lavaux 49, CH1095 Lutry, Switzerland

**Keywords:** SARS-CoV-2 vaccines, myocarditis, pre-existing comorbidities

## Abstract

COVID-19 vaccines have been widely used to reduce the incidence and disease severity of COVID-19. Questions have lately been raised about the possibility of an association between COVID-19 vaccines and myocarditis, an inflammatory condition affecting the myocardium, or the middle layer of the heart. Myocarditis can be caused by infections, immune reactions, or toxic exposure. The incidence rate of myocarditis and pericarditis was calculated to be 5.98 instances per million COVID-19 vaccine doses delivered, which is less than half of the incidences after SARS-CoV-2 infection. Myocarditis rates in people aged 12 to 39 years are around 12.6 cases per million doses following the second dose of mRNA vaccination. Adolescent men are more likely than women to develop myocarditis after receiving mRNA vaccines. The objectives of this systematic review and meta-analysis are to find out how often myocarditis occurs after receiving the COVID-19 vaccine, as well as the risk factors and clinical repercussions of this condition. Nevertheless, the causal relationship between vaccination and myocarditis has been difficult to establish, and further research is required. It is also essential to distinguish between suggested cases of myocarditis and those confirmed by endomyocardial biopsy.

## 1. Introduction

Myocarditis is an inflammatory disorder that affects the myocardium, or middle layer of the heart, and can be caused by infections, immunological responses, or exposure to toxins. The International Classification of Diseases (ICD) codes in hospital discharge documentation between 1990 and 2013 were used in the Global Burden of Disease study to estimate the incidence of myocarditis at 22 cases per 100,000 patients [1].

Myocarditis has been described by different clinical manifestations such as acute, fulminant, subacute, and chronic. Acute or active myocarditis should rather be described as clinically suspected myocarditis with an acute presentation [2]. A severe and rapidly progressing manifestation of clinically suspected myocarditis with acute presentation called fulminant myocarditis causes cardiogenic shock and requires the use of inotropes or mechanical circulatory support. If there is evidence of prior active myocarditis, subacute myocarditis can also be classified as healing myocarditis. Subacute myocarditis is characterized by a continuous myocardial injury caused by a chronic or repeated stimulus for myocardial inflammation. The time between the beginning of symptoms and the diagnosis of subacute myocarditis might also be greater than or equal to one to three months. There is an overlap with the description of subacute myocarditis since the disease process is believed to be chronic inflammatory cardiomyopathy when symptoms last for more than a month [3].

Myocarditis is histopathologically defined by an inflammatory cellular infiltration, which can be localized or widespread, whether there is cardiac myocyte damage or not [4]. Overall, clinically suspected myocarditis can be difficult to diagnose because the 2013 European Society of Cardiology guidelines are not always fulfilled, affects people of various ages, and symptoms commonly manifest between the ages of 20 and 50 [5]. The most frequent symptoms are chest discomfort (in 85–95% of patients) and dyspnea (19–49% of cases) [6]. Since prodromal symptoms are present in up to 80% of cases with clinically suspected myocarditis with acute presentation, patients may have previously had gastrointestinal, respiratory, or flu-like symptoms. However, even though 26% of patients with clinically suspected myocarditis with acute presentation have aggravating symptoms such as left ventricular systolic dysfunction, persistent ventricular arrhythmias, or a fulminant presentation with low cardiac output syndrome, an examination may reveal no notable abnormalities [7]. Moreover, a large number of diagnosed cases of myocarditis described after COVID-19 vaccinations are affected by diagnostic inaccuracy and over-diagnosis and should be distinguished from cases confirmed by endomyocardial biopsy (EMB) [8]. Therefore, the temporal association between COVID-19 vaccination and myocarditis does not imply the existence of a causal relationship.

**Figure 1:**
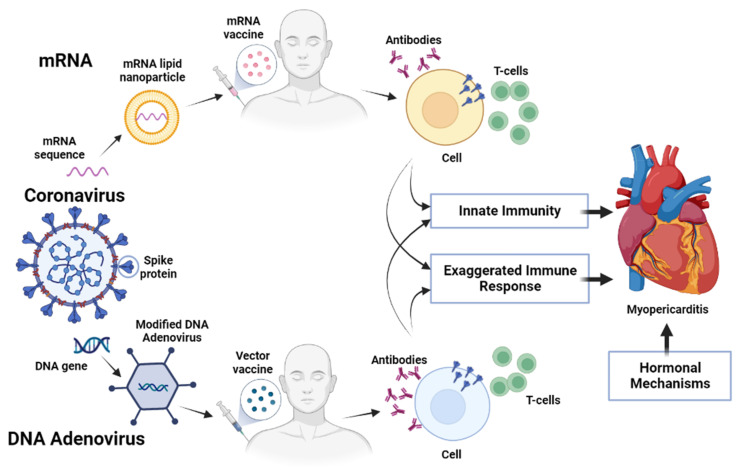
Possible action of COVID vaccines and development of myopericarditis [34].

Since the beginning of the COVID-19 pandemic, initially identified in the city of Wuhan in China in December 2019 and rapidly spreading throughout the world, myocardial inflammation has been suspected. The etiology is not fully understood, but the two leading hypotheses suggest that the ACE2 receptor directly contributes to the disease and that a hyperimmune response may also result in an isolated presentation of COVID-19-mediated myocarditis (Figure 1). It is unknown how frequently COVID-19-mediated myocarditis occurs and how it affects prognosis [9]. High troponin levels have been associated with higher mortality in patients with COVID-19, according to previous research. Unusual troponin levels, however, may not always indicate clinically suspected myocarditis with acute presentation [10,11] and require a clinical scenario for myocarditis diagnosis. Enhanced troponin levels are frequently seen in myocarditis. However, it can be difficult to distinguish between myocardial damage and myocarditis [12]. In a proper clinical setting, a large elevation of troponin may be suggestive of clinically suspected myocarditis with acute presentation in the absence of myocardial ischemia. Although 5 out of 104 COVID-19 patients tested positive for SARS-CoV-2 gene-specific sequences in EMBs [13], the precise causes of cardiac damage after SARS-CoV-2 infection are still largely unclear, and despite the criteria form the Working Group of the European Society of Cardiology 2013, over-diagnosis of myocarditis cases is not uncommon [14].

However, the introduction of various COVID-19 vaccines has led to a considerable decrease in COVID-19-related morbidity and death around the world, and all licensed COVID-19 vaccines have shown advantages that outweigh the possible dangers among various age groups [15,16]. During the COVID-19 pandemic, a prevalence of 2.4–4.1 cases of myocarditis among 1000 hospitalized patients has been estimated [17,18,19,20]. Although cardiac complications are globally rare (20 cases/100,000 per year) [1,21], they are more closely associated with SARS-CoV-2 infections than with COVID-19 vaccinations [22]. Myocarditis may directly relate to SARS-CoV-2 infection and/or bacterial co-infections and superinfections [23,24,25], which propagate myocarditis manifestations through direct interaction between the SARS-CoV-2-proteome and the cardiac cellular ACE receptor [26,27,28]. A systematic study reported by Gao et al. [29] with 58,620,611 subjects showed that immunization against COVID-19 was associated with an increased incidence of myocarditis or pericarditis (RR = 2.04; 95% CI = 1.33, 3.14). Furthermore, persons who got the second dose of COVID-19 vaccination had a higher risk of myocarditis or pericarditis compared to those who only received the first dose (RR = 4.06; 95% CI = 2.08, 7.92).

Previously, we published a review on the possible association between COVID-19 vaccines and non-communicable diseases (NCDs) [30], which only briefly covered myocarditis. Here, we aim to present data on COVID-19 vaccines (mRNA-based, adenovirus-based, and inactivated-virus-based vaccines) and their possible impact on myocarditis development. Additionally, we present other factors which can contribute to myocarditis development such as pre-existing disorders, age, sex, and genetic predisposition.

## 2. Possible Molecular Mechanisms of COVID-19-Vaccine-Induced Myocarditis

Autoantibodies and hormone-related variables play a role in the sex differences reported in both COVID-19 mRNA-vaccine-related myocarditis and non-COVID-19 viral myocarditis. Vaccine-induced spike (S)-protein IgG antibodies block the attachment of SARS-CoV-2 to host cells through S protein binding to ACE2 and thereby kill the virus. Immune reactivity to mRNA, antibodies to SARS-CoV-2 S glycoprotein cross-reacting with cardiac contractile proteins, and hormonal variations are the three primary mechanisms by which COVID-19 mRNA vaccines may produce hyper immunity [31]. The immune system may recognize the mRNA as an antigen, triggering pro-inflammatory cascades and immunological pathways in the heart. Although nucleotide changes to mRNA diminish its innate immunogenicity, the immunological response to mRNA may nevertheless cause the activation of an abnormal innate and acquired immune response, explaining why mRNA vaccines elicit a higher immune response than other types of COVID-19 vaccines. Antibodies designed against the SARS-CoV-2 S glycoprotein may cross-react with structurally comparable human protein sequences, such as cardiac-myosin heavy chain [31]. However, based on a recent study, increased myocarditis occurrence was not mediated by a cross-reactive adaptive immune response after the COVID-19 vaccine-based expression of the SARS-CoV-2 S protein [32]. Furthermore, given the higher prevalence of COVID-19 mRNA-vaccine-related myocarditis in male patients, variations in signaling may play a role in the pathophysiology of COVID-19 mRNA-vaccine-related myocarditis [33]. However, the key message is that myocarditis development after COVID-19 vaccinations is rare and almost always of a benign nature [33].

## 3. Myocarditis and Cancer

According to a case report, a 62-year-old patient with lung adenocarcinoma developed myocarditis after vaccination with the mRNA-1273 vaccine [35]. In September 2019, she was diagnosed with stage IV lung cancer with bilateral lung and lymph node involvement. Twenty-four hours after the third mRNA-1273 vaccine dose, the patient was admitted to the emergency room with clinically suspected myocarditis with acute presentation and signs of significant left ventricular (LV) failure in cardiogenic shock. She required vasoactive support, non-invasive mechanical breathing, corticotherapy, immunoglobulins, and later ventricular support with Impella, resulting in clinical improvement after three days [35]. Magnetic resonance imaging (MRI) of the heart revealed indications of widespread myocardial edema consistent with acute myocarditis. This is an unusual and debatable example of fulminant myocarditis, which occurred after COVID-19 vaccination in a patient with metastatic lung cancer. Therefore, comprehensive monitoring of heart function is recommended for vaccinated cancer patients. This case study demonstrates the importance of future research to monitor adverse responses in vaccinated cancer patients as well as the potential interactions of vaccines with antineoplastic medicines [35].

## 4. Myocarditis Related to Genetics, Age, and Sex

Myocarditis seems to have a greater prevalence in males than in females [36]. Thus, a 2.5:1 (male/female) ratio of myocarditis was reported after a poliomyelitis outbreak that occurred in Minnesota in 1946 [37], and another study on coxsackieviruses in 1968 showed an incidence of myocarditis in males vs. females of about 72% [38]. In a survey of 164 adolescent and adult cases of coxsackievirus B myopericarditis in 1980, 67% of the patients were men [39]. Furthermore, in a recent analysis of 322 consecutive patients who were hospitalized with the diagnosis of a clinically suspected myocarditis with acute presentation, 84% were men [40]. Enterovirus or human herpesvirus infections generally cause a more severe form of myocarditis at a younger age and in males [33].

Recent studies suggest that the interaction of environmental factors (mainly viruses) with the genetic background can potentially have an impact on myocarditis development and susceptibility [41]. Thus, there may be a genetic predisposition toward the development of the disease [42], although our current knowledge is too preliminary to make concrete conclusions. A myocardium that shows genetic variations in structural proteins is more susceptible to myocardial targeting by pathogenic microorganisms. Myocarditis can be associated with an immune genetic background that increases the likelihood of developing clinically suspected myocarditis with acute presentation after viral infections, such as genetic variants in genes encoding HLA factors and, in a minority of patients, genetic variants in genes encoding desmosomal, cytoskeletal, or sarcomeric proteins [43]. The course of events leads one to believe that a genetic change in the desmosome has produced the ideal conditions for an infectious agent [42]. Compared to patients with clinically suspected myocarditis with acute presentation without pathogenic desmosomal gene variants, patients with clinically suspected myocarditis with acute presentation and evidence of pathogenic desmosomal gene variants have a higher rate of adverse cardiovascular events [44]. The most recent research emphasizes the potential prognostic value of some pathogenic mutations that might leave a myocardium susceptible to myocardial inflammation and the emergence of chronic cardiomyopathy [41].

## 5. COVID-19 Vaccines and Myocarditis in Children, Adolescents, and Young Adults

One of the potential complications of the mRNA-based COVID-19 vaccines (BNT162b2, Pfizer–BioNTech) and mRNA-1273 (Moderna)) in adolescents and young adults is myocarditis [45,46,47,48]. Regarding the influence of age on myocarditis, a report revealed that the disease leading to hospital admission is relatively rare in children [49]. However, the occurrence of myocarditis increases with age. Moreover, the study indicated that there are no differences related to sex in the risk of myocarditis during the first 6 years of life. However, boys show a significantly higher risk at ages 6 to 15 years [49].

Although myocarditis has been uncommon for other viral vaccines, postvaccination myocarditis has been described in adolescents and young adults after mass vaccinations with the BNT162b2 vaccine. For example, acute symptomatic myocarditis was suspected in seven adolescents since no EMB was performed [45]. The seven patients recovered, and no causal relationship between vaccine administration and myocarditis could be established. On 23 June 2021, CDC’s Advisory Committee on Immunization Practices (ACIP) reviewed available data and concluded that the benefits of COVID-19 vaccination for individuals and the population in general outweigh the risks for myocarditis and recommended the continuation of the vaccination of children 12 years of age and older [50]. In the same study, it was indicated that although adverse events were reported in approximately one per 1000 vaccinees from 8.9 million adolescents according to the VAERS (Vaccine Adverse Event Reporting System) between 14 December 2020 and 16 July 2021, in the US, 90% of the cases were non-serious conditions. Furthermore, no myocarditis-related deaths were reported [50]. These conclusions are in line with the preauthorization trials for the BNT162b2 vaccine among people aged 12–25 years [51].

In the US, as of 11 June 2021, adolescents aged 12–17 years old had received 4,229,597 doses of the BNT162b2 vaccine [52], and by 1 July 2021, 22.1% of adolescents 12–15 years of age had been fully vaccinated [53]. On 11 June 2021, of the 2,189,726 and 2,039,871 doses administered to adolescent females and males, respectively, there were 19 (female) and 128 (male) cases of myocarditis/pericarditis, with the risk of developing myocarditis/pericarditis being higher after the second dose (ACIP), [54].

The prevalence rates of myocarditis potentially associated with COVID-19 vaccination in adolescents show a wide range of variation. The overall incidence of myopericarditis after the second dose of the BNT162b2 vaccine was estimated to be approximately 0.01% and 0.008% in adolescents 12–15 years and 16–17 years, respectively [55]. According to the US CDC, myocarditis/pericarditis rates are approximately 12.6 cases per million doses after the second dose of mRNA vaccines among individuals 12 to 39 years of age [18]. Based on the analysis of VAERS data from 11 December 2020 to 13 August 2021, an incidence rate of 5.98 (95% CI = 5.73–6.24) cases per million doses administered was estimated for adverse events of myocarditis and pericarditis after COVID-19 vaccination. A higher incidence rate was observed in adolescents and especially after the administration of the second dose of mRNA vaccines [56]. Adolescent males are more frequently affected by myopericarditis after the administration of mRNA COVID-19 vaccines than females, with a reported incidence of 32.4 and 4.2 per million doses in males and females, respectively [18,46,57].

In Danish adolescents, the incidence of myopericarditis after COVID-19 vaccination among males 12–17 years of age was 97 per million compared to 63 per million in the US [58]. In the US, the incidence of myocarditis/pericarditis after the second dose of the mRNA vaccine in male patients 12–15 and 16–17 years of age was 162.2 and 93.0 per million, respectively [59]. Although favorable risk-benefit of two doses of the BNT162b2 vaccine was seen in non-immune girls with a comorbidity, it was not the case in boys with prior SARS-CoV-2 infection and no comorbidities. Thisstrongly supports individualized pediatric COVID-19 vaccination strategies [59]. In South Korea, among 12th-grade students (high school seniors) who received the BNT162b2 vaccine, the rate of myocarditis and/or pericarditis was 1.8 per 100,000 (95% C.I. 0.8–3.5) among first-dose recipients and 4.3 per 100,000 (95% CID 2.6–6.7) in second-dose recipients [60]. Curiously, children, 5–11 years of age showed substantially lower reported rates of myocarditis after the second dose of the BNT162b2 vaccine than those observed among adolescents 12–15 years of age [61]. Similarly, in Danish children, the risk of myopericarditis was 4.8 (95% confidence interval (CI), 0.1 to 26.8) per 1,000,000 vaccinated individuals 5–11 years of age, which was significantly lower than the risk in individuals 12–17 years of age of 57.4 (95% CI, 32.1 to 94.7) per 1,000,000 [62].

Despite a rather broad variability in the reported incidents of postvaccination myocarditis in adolescents, most of the studies agreed that rates of myocarditis are higher after the second dose of the BNT162b2 vaccine, that males are affected much more frequently than females, that the penetrance of myocarditis peaks in the 16–17-year-old vaccine recipients, that the reporting rate is highest in individuals with a short (less than 30 days) interval between doses, and that in most cases, the COVID-19-vaccination-associated symptoms are mild and followed by rapid recovery with conservative treatment. It is important that despite its overall low incidence, clinicians should consider myocarditis and pericarditis as probable diagnosis in patients with suggestive symptoms after vaccine administration.

## 6. COVID-19 mRNA Vaccines and Myocarditis

In the general population, myocarditis and pericarditis have been found in about 0.02% and 0.01% of vaccine recipients, respectively, in clinical trials for the BNT162b2 vaccine. Similarly, clinical trials for the mRNA-1273 vaccine revealed that about 0.03% of vaccine recipients developed myocarditis and about 0.01% developed pericarditis [61,63]. Most cases of myocarditis have been reported in case-report studies. The main findings are described below and summarized in Table 1. As described in the previous section, the majority of the patients were young men, and they complained of chest pain and showed elevated cardiac troponin levels two to three days after receiving the second dose of the mRNA vaccine (BNT162b2 and mRNA-1273) [64]. However, there is minimal evidence that the risk of adverse effects, such as myocarditis and pericardial effusion, increases with subsequent doses of the vaccine. Myocarditis symptoms were evident on cardiac MRIs and electrocardiograms in most of these instances. For most patients, the medication was unnecessary as symptoms faded and diagnostic markers improved [65]. A small number of Israeli men in their early twenties developed myocarditis in April 2023 after receiving the SARS-CoV-2 BNT162b2 and mRNA-1273 vaccines [66,67]. Hundreds of observational studies conducted in Asia [68,69,70], Europe [71,72,73], and the US [67,74] have been published.

The occurrence of myocarditis, pericarditis, and cardiac arrhythmias was investigated in people who received the COVID-19 vaccine (BNT162b2 or mRNA-1273) and in people who tested positive for SARS-CoV-2 in England [75]. The risk of myocarditis was shown to increase after the first dose of the mRNA-1273 or BNT162b2 vaccine. The risk of myocarditis was higher in SARS-CoV-2-positive individuals. Furthermore, people who tested positive for SARS-CoV-2 had an increased risk of pericarditis and cardiac arrhythmias, which was not the case in vaccinated people, except for those who received a second dose of the mRNA-1273 vaccine [25,75]. Myocarditis was more frequently detected after immunization with mRNA vaccines in individuals younger than 40 years of age [71,76]. In a study that included more than 38 million participants, 3576 cases of myocarditis, pericarditis, and cardiac arrhythmias that led to hospitalization or death were detected [71,77]. The first dose of BNT162b2 caused one extra myocarditis event per one million people 1–28 days postvaccination. On the contrary, six additional cases of myocarditis events per one million people were seen after vaccination with the mRNA-1273 vaccine. However, these numbers are low compared to the 40 additional cases of myocarditis in individuals who tested positive for SARS-CoV-2. Enhanced risks of pericarditis and cardiac arrhythmias were also seen in SARS-CoV-2-positive individuals, but not in persons vaccinated with either the BNT162b2 or mRNA-1273 vaccine, except for an increased risk of arrhythmia after the second dose of the mRNA-1273 vaccine.

**Table 1:** Examples of myocarditis after vaccination with mRNA-based COVID-19 vaccines.

Vaccine	Cases (N)	Frequency	Number of Biopsy Proven Myocarditis Cases	Findings
BNT162b2	54	2.13 case/10,000	N/A	A small number of males in Israel [78]
BNT162b2 mRNA-1273	42,200,614	3/1 million 12/1 million	N/A N/A	Increased risk of myocarditis after the first dose [71] Increased myocarditis risk after the first dose [71]
BNT162b2 mRNA-1273	Not declared	14/1 million 101/1 million	N/A N/A	Increased risk of myocarditis after the second dose [71] Increased risk of myocarditis after the second dose [71]
BNT162b2	Not declared	13/1 million	N/A	Increased risk of myocarditis after the third dose [71]
BNT162b2	160 out of 1533	0.57 confidence intervals (CIs)	N/A	Increased risk of myocarditis risk after two doses [68]
BNT162b2 mRNA-1273	48 21	1.4/100 K 4.2/100 K	N/A N/A	Increased risk of myocarditis in females [79] Increased risk of myocarditis risk in 12–39-year-olds [79]
BNT162b2 mRNA-1273	Not declared	5.55/100 K 18.4/100 K	N/A N/A	Increased risk of myocarditis after the second dose [80] Increased myocarditis risk after the second dose [80]
BNT162b2	20	4.8/100 K	2	Increased risk in 16–19-year-old males in Israel [81]
BNT162b2 mRNA-1273	7 16	7/23 16/23	N/A N/A	Myocarditis in previously healthy males after the second dose [77]. Myocarditis in previously healthy males after the second dose [77]

N/A, not available.

A case–control study on 1533 healthy people served as controls for 160 people diagnosed with cardiac problems [68]. The BNT162b2 vaccine was associated with a 95% confidence interval (CI) of 0.36 for the same outcome. According to the findings, those who were vaccinated with BNT162b2 were less likely to develop carditis than those who did not (OR, 3.57 (CI, 1.93 to 6.60)). Compared to females, males had a higher risk (OR, 4.68 (CI, 2.25 to 9.71)), as did adults (2.41 (CI, 1.18 to 4.90)) and adolescents (2.41 (CI, 1.18 to 4.90) (OR, 13.79 (CI, 2.86 to 110.38))). When the BNT162b2 vaccine was administered twice, the risk increased (OR, 9.29 (CI, 3.94 to 21.91)) [68]. However, the report was based on a limited sample size.

Myocarditis and myopericarditis rates were also compared in vaccinated and unvaccinated Danes in a separate cohort study [78]. Myocarditis and myopericarditis were shown to be much more common in those who had received the mRNA-1273 vaccine, particularly those between the ages of 12 and 39 years and beingfemales. On the other hand, a significant increase in risk was only seen in females for the BNT162b2 vaccine [79].

The risk of myocarditis was highest in young men (16–24 years old) after a second dose of the SARS-CoV-2 mRNA vaccine, according to a large cohort study of 23.1 million participants from four Nordic countries [71]. The study indicated that after the second dose of the BNT162b2 vaccine, 4–7 additional cases of myocarditis were observed per 100,000 vaccinated individuals. In the case of the mRNA-1273 vaccine, there were 9–28 additional cases of myocarditis per 100,000 vaccine recipients. However, the presence of rare cases of myocarditis in vaccinated individuals must be related to the benefits of vaccination preventing severe COVID-19 disease, as shown in this study [80]. A retrospective study investigated the incidence of myocarditis in individuals vaccinated with the BNT162b2 vaccine in Israel [68]. A total of 304 individuals had symptoms of myocarditis among the more than 9 million individuals monitored.

Additional case reports within the US Military Health System reported that 23 men with a median age of 25 years were diagnosed with myocarditis after receiving the BNT162b2 (7 persons) or mRNA-1273 (16 persons) vaccine [79]. None of the individuals had a history of heart disease, but the underlying causes, such as infection, ischemia, or autoimmunity, were never determined. Electrocardiograms and echocardiograms showed aberrant results in 19 (83%) patients. Furthermore, four patients showed reduced LVEF (17%). Within a week after receiving the BNT162b2 vaccine, 16 patients reported improvement in cardiac symptoms, while 7 individuals continued to experience chest discomfort. Although the small sample size, the dependence on passive surveillance, and incomplete testing could not confirm a causal association between vaccinations and myocarditis, it demonstrated the importance of monitoring potential adverse events of mass vaccinations [77].

Myocarditis and pericarditis were discovered at a rate higher than average after BNT162b2 or mRNA-1273 vaccinations based on the active surveillance of large healthcare databases [82]. Individuals 18–25 years of age were at the highest risk after receiving their second dose of either vaccine. There was no statistically significant difference between the mRNA-1273 and BNT162b2 vaccines in terms of the risk of myocarditis or pericarditis. The results are consistent with those of previous investigations using the same passive monitoring system in Europe [83], Canada [84,85], the UK [71,78,83,86], Denmark [58,62,87], and the US [50,88,89]. Risk analyses by the FDA and the Advisory Committee on Immunization Practices show that the advantages of COVID-19 vaccination still clearly outweigh the risks, even with the increased risk of myocarditis or pericarditis [75]. After receiving reports of an elevated risk of myocarditis and pericarditis, especially in younger men, the FDA revised the Emergency Use Authorization Fact Sheets for the BNT162b2 and mRNA-1273 vaccines [90].

Sixteen patients were diagnosed with myopericarditis after receiving the second dose of the BNT162b2 vaccine [91]. This study sheds light on the prevalence and clinical presentation of myopericarditis after the administration of the BNT162b2 vaccine. It also emphasizes the importance of monitoring symptoms emerging after vaccination to establish whether there is a causal relationship, and if so, to design the best treatment for vaccine-associated myopericarditis [91].

A systematic study and meta-analysis of the incidence of myopericarditis following vaccination with COVID-19 vaccines and non-COVID-19 vaccines included a total of 22 studies and more than 405 million vaccine doses [87]. There were no statistically significant differences in the overall incidence of myopericarditis between people who received COVID-19 vaccines and those who received non-COVID-19 vaccines. However, compared to COVID-19 vaccinations, the incidence of myocarditis was significantly higher after vaccinations against smallpox, but not against the influenza virus vaccinations. Other factors affecting the incidence of myopericarditis are sex, where males have a significantly greater risk than females, and age, in which case, people younger than 30 years are more prone to develop myopericarditis than people older than 30 years. Moreover, the risk of myopericarditis is higher for vaccinations with mRNA vaccines than non-mRNA vaccines, and the second vaccine dose poses a bigger risk than the first dose [87]. The study surveyed 107 pediatric facilities in the US and 57 completed the survey, 15 of whom reported having a vaccine-associated myopericarditis (VAM) treatment regimen in place. The study found that arrhythmias were common, but fatal complications were rare. The incidence of suspected myopericarditis temporally associated with COVID-19 mRNA vaccinations in females and males 12–17 years of age was estimated to range from 4.2 to 32.4 per million administered doses, respectively. The results confirmed previous reports that significant, potentially fatal adverse events are rare in adolescents with VAM, and all patients made a full recovery, with a positive short-term prognosis [46]. It is also important to point out that suspected myocarditis cases were not confirmed by EMBs.

Non-steroidal anti-inflammatory drugs (NSAIDs), intravenous immunoglobulin (IVIg), and prednisone were administered to the patients. Treatment with IVIg and/or corticosteroids may be an option, but only for selected EMB-confirmed virus-negative myocarditis cases. It is recommended that medical professionals consider myocarditis as a possible diagnosis for patients who complain of chest pain following COVID-19 vaccination [45].

## 7. Recombinant Adenovirus Vector-Based COVID-19 Vaccines and Myocarditis

Although in 2021, it was suggested that recombinant adenovirus-vector-based vaccines are not associated with myocarditis [92,93], in more recent studies, myocarditis has been reported after vaccination with COVID-19 vaccines [75]. Several mechanisms have been indicated to trigger myocarditis as adenoviruses can cause clinically suspected myocarditis with acute presentation [39,94]. Adenoviruses can infect cardiomyocytes by binding to the coxsackie and adenovirus receptor (CAR), which induces direct myocardial injury and triggers an uncontrolled immune response [95]. Moreover, although the similarity between the SARS-CoV-2 S protein and human antigens such as α-myosin and actin could lead to cross-reactivity [31], a recent study indicated that this is not the case [32]. Dysregulated host responses are also triggered by repeated antigen exposure, which can lead to polyclonal B-cell expansion, immune complex formation, and inflammation [94]. Furthermore, another possible mechanism for myocarditis after SARS-CoV-2 infection or COVID-19 vaccination is related to anti-idiotype antibodies [96].

In a self-controlled case study series in 16-year-old and older individuals, it was estimated that two extra cases of myocarditis events per 1 million persons occurred within 1–28 days after vaccination with the ChAdOx1 nCoV-19 adenovirus-based vaccine in the UK [71]. However, this should be compared to 40 additional cases of myocarditis events per 1 million people in SARS-CoV-2-positive individuals.

In another series of case studies in 13-year-old and older individuals, the association between vaccination and myocarditis was evaluated based on age and sex [97]. The risk of myocarditis increased 1–28 days after vaccination with the ChAdOx1 nCoV-19 vaccination with an incidence ratio of 1.33, which was significantly lower than in SARS-CoV-2-positive individuals who were 11.14 and 5.97 before and after vaccination, respectively. The association between vaccination and myocarditis was stronger in men under the age of 40 years. In another case report study, myocarditis was diagnosed in a 32-year-old female after receiving the first dose of the ChAdOx1 nCoV-19 vaccine [97]. However, it was not possible to confirm a direct association between vaccination and the development of myocarditis. In addition, acute fulminant perimyocarditis was discovered in a 44-year-old woman after the first dose of the ChAdOx1 nCoV-19 vaccine [94]. Her symptoms gradually disappeared, and she recovered completely. Although vaccine-related myocarditis was suggested, the causal relationship could not be confirmed. In the context of the Ad26.COV2.S vaccine, requiring only a single immunization, the induction of lymphohistocytic myocarditis was reported, leading to the death of the patient [98]. However, this case is quite controversial, and there is no proof of a causal association between the vaccination and myocarditis development. Furthermore, the absence of biopsy-confirmed diagnosis and previous steroid treatments cannot exclude the concept that the death was not caused by myocarditis and that it was unrelated to vaccination. In another case-series study, an individual showed evidence of myocardial injury after receiving the Ad26.COV2.S vaccine, but the symptoms disappeared by the time of the discharge from the hospital [99]. Again, only the temporal association between COVID-19 vaccination and myocarditis could be confirmed.

## 8. Inactivated Virus and Protein Subunit COVID-19 Vaccines and Myocarditis

As inactivated viral COVID-19 vaccines also contain viral structural proteins and RNA, non-adaptive immune responses could be induced, leading to the production of inflammatory responses and the development of myocarditis and/or lethal fulminant myocarditis (Figure 1) [100,101,102,103]. Molecular mimicry of the S protein and some unknown cardiac protein has been suggested as a mechanism [104,105]. However, recent findings indicate that it is not the case [32].

A case–control study in a predominantly Chinese ethnic population in Hong Kong compared the incidences of carditis after vaccinations with either the BNT16b2 mRNA vaccine or the CoronaVac inactivated virus vaccine in individuals 12 years of age or older [68]. The estimated incidence of carditis per 100.000 persons for the CoronaVac and BNT162b2 vaccines was 0.31 (95% CI, 013–0.66) and 0.57 95% CI, 0.36–0.90), respectively [68], indicating a similar magnitude of association between carditis and the CoronaVac and BNT162b2 vaccines. A case of perimyocarditis was reported in an older woman subjected to a heterologous vaccination strategy receiving a BNT162b2 booster vaccination after three doses of the CoronaVac vaccine [106]. Although myocarditis/pericarditis is more common in males younger than 40 years old, anecdotal cases of fulminant myocarditis have been reported in elderly individuals after BNT162b2 vaccination [107]. In a comparative study in Hong Kong, it was demonstrated that the perimyocarditis rates were significantly higher after SARS-CoV-2 infection than after vaccination. Of a total of 2,811,500 doses of the CoronaVac (37.05%) and 4,776,700 doses of the BNT162b2 (62.95%) vaccines administered, only 42 cases of perimyocarditis were detected [108]. Of these, 41 individuals received the BNT162b2 vaccine and only 1 person the CoronaVac vaccine. A comparison of vaccinations in Israel, the UK, and the US showed similar rates of myocarditis. Although the manifestation of allergic/eosinophilic symptoms has been described in COVID-19 patients [109], no cases of hypereosinophilic syndrome have been reported after the administration of inactivated virus vaccines [110].

In a self-controlled case-series study in Malaysia, no increased risk of myocarditis/pericarditis was reported after the administration of the CoronaVac, BNT162b2, or ChAdOx1 nCoV-19 vaccines [111]. Seven cases of myocarditis/pericarditis were detected after the first dose of the CoronaVac vaccine and two cases after the second dose. Based on another self-controlled case-series study in Hong Kong, it was concluded that individuals receiving three doses of the CoronaVac vaccine showed no increased risk of adverse events after the first and the second doses [112]. Only a significantly increased risk of anaphylaxis was observed after the second dose of the vaccine. It was also noted that as older age is associated with a poorer outcome of SARS-CoV-2 infection, the benefits of CoronaVac vaccinations outweigh the risks.

In a case study, it was reported that a 34-year-old female developed fulminant myocarditis 8 days after receiving the COVID-19 protein subunit vaccine ZF2001, which had been conditionally approved in China [113]. The patient showed elevated levels of myocardial enzyme and biomarkers and the severe infiltration of lymphocytes and monocytes in the left and right ventricular walls, which was associated with myocyte degeneration and necrosis, eventually leading to her death [113]. A causal relationship between vaccination and myocarditis could not be established, and further research is required. Table 2 represents reported carditis cases in individuals immunized with inactivated COVID-19 and protein subunit vaccines between January 2020 and January 2023.

## 9. Conclusions

Myocarditis has returned to the spotlight during the COVID-19 pandemic. Hospitalized COVID-19 patients have a prevalence of myocarditis of 2.4–4.1 cases per 1000 cases. Myocarditis may have a direct connection to SARS-CoV-2 infection and/or bacterial co-infections and super-infections [23,24], resulting in myocarditis via direct interaction with cardiac cellular receptors, such as ACE2 in the case of SARS-CoV-2. Some studies have indicated that COVID-19 immunization may increase the risk of myocarditis. However, in the great majority of cases, no EMB data are available, making the confirmation of the right myocarditis diagnosis questionable. The primary objective of this review was to provide information on the potential causal association of mRNA-, adenovirus- and inactivated-whole-virus-based COVID-19 vaccines and myocarditis.

The safety and efficacy of COVID-19 mRNA vaccines have been examined in extensive clinical studies. Myocarditis and pericarditis have been mentioned in rare cases as adverse effects in several investigations. In clinical studies for the BNT162b2 vaccine, myocarditis and pericarditis were found in roughly 0.02% and 0.01% of vaccine recipients, respectively. The mRNA-1273 (Moderna) vaccine also showed that about 0.03% of recipients experienced myocarditis, and about 0.01% experienced pericarditis in clinical trials [36,37].

Despite the claim in 2021 that recombinant adenovirus-vector-based vaccinations are not linked to myocarditis [92,93], myocarditis has been documented in more recent trials after immunization with adenovirus-based COVID-19 vaccines [75]. As adenoviruses can induce clinically suspected myocarditis with acute presentation, a number of mechanisms have been suggested to initiate myocarditis [39,94]. However, only a temporal relationship between COVID-19 vaccines and myocarditis has been proven so far, and additional studies are needed to determine whether a causal relationship exists.

Due to the inclusion of viral structural proteins and RNA in inactivated viral COVID-19 vaccines, non-adaptive immune responses can be triggered, which might result in the overproduction of inflammatory responses and the development of myocarditis and/or deadly fulminant myocarditis [100,101,102,103]; however, a causal relationship between vaccination and myocarditis could not be established, and further research is required.

In summary, the current COVID-19 vaccines have clearly demonstrated efficacy and undoubtedly proved that the benefits overweigh the risks by a big margin. However, it cannot be denied that adverse events, although at a low frequency, including the development of myocarditis, have been documented in vaccinated individuals. Due to the global administration of more than 13.3 billion COVID-19 vaccine, doses it is not unexpected for adverse events to occur. For this reason, it is important that clinicians monitor any symptoms developed after vaccinations with current COVID-19 vaccines.

## Figures and Tables

**Table 2:** Carditis reported in published peer-reviewed articles in individuals immunized with inactivated COVID-19 and protein subunit vaccines between January 2020 and January 2023 (uncontrolled hypertension, diabetes, cardiovascular disease, liver and kidney disease, malignancy, autoimmune diseases, and other comorbidities were excluded). All patients were discharged from hospital, except a female who passed away [112].

Patients (N)	Age	Gender M/F	Presence of Coronary Artery Disease	Symptoms	Biopsy Confirmation of Virus-Negative Myocarditis (ESC 2013 Criteria)	Vaccine Type	Dose	Time Post-Infection	Ref.
1	50	Male	N/A	Acute onset of chest discomfort at rest	N/A	CoronaVac	1st	12 h	[114]
1	49	Male	Yes	Retrosternal chest pressure at rest	N/A	CoronaVac	2nd	18 h	[114]
1	41	Female	No	Flushing, palpitation, lip and tongue swelling, shortness of breath, and chest pain	N/A	CoronaVac	1st	15 min	[115]
1	72	Female	N/A	Chest pain and dyspnea Interstitial fibrosis with patchy myofibrillar loss without significant lymphocyte infiltration	no	BNT162b2 booster 6 months after 3 CoronaVac doses	Booster	2 days	[106]
60	>18	36/24	No	Carditis cases represented background incidence of 0.31 per 100.000 doses, absolute risk increase of 0.26 per 100,000 doses, 0.17 per 100.000 doses after 1st dose, and 0.4 per 100.000 doses after 2nd dose	N/A	CoronaVac	1st and 2nd 39 people received 2nd dose	Screening for 14–30 days	[68]
2	63, 57	1/1	no/no	Fulminant myocarditis	yes/yes	Inactivated BBIBP-CorV (Vero cell) Sinovac	1st	1/4 days	[116]
1	29	Female	No	Fulminant myocarditis	N/A	BIBP (Sinopharm)	2nd	3 months	[117]
1	72	Male	N/A	Constrictive pericarditis, exertional dyspnea, heart failure Unusual fatigue, decreased appetite abdominal swelling as concurrent symptoms but no chest pain, orthopnea, or abdominal pain	N/A	Inactivated BBIBP-CorV (Vero cell) Sinovac	3rd	8 days	[104]
1	Early 20s	Male	N/A	Myocarditis, deep cramping pain Endocardial biopsy showed active lymphocytic myocarditis with infiltrates and focal myocyte injury	yes	COVAXIN	1st	2 months	[118]
1	33	Male	N/A	F Hyper-eosinophilia; multiorgan involvement of skin, subcutaneous tissue, and myocardium; shortness of breath exertion, even while talking	N/A	COVAXIN	1st	25 days	[110]
1	34	Female	N/A	Fulminant myocarditis Postmortem examination showed a bilateral pleural cavity Biopsy revealed mild atherosclerotic stenosis in left anterior descending coronary artery and right coronary artery Multi-focal inflammatory infiltration	N/A	ZF2001 RBD-subunit	1st	8 days	[113]
1	13	Male	N/A	Myocarditis Chest pain on day 5, lasting for 2 days	N/A	Inactivated BBIBP-CorV	1st	5 days	[119]

N/A, not available.

## Data Availability

Not applicable.
